# Allogeneic haematopoietic stem cell transplantation with decitabine-containing preconditioning regimen in TP53-mutant myelodysplastic syndromes: A case study

**DOI:** 10.3389/fonc.2022.928324

**Published:** 2022-07-18

**Authors:** Yuxin Wang, Yao Sun, Jing Xie, Jiangwei Hu, Na Liu, Jianlin Chen, Botao Li, Sanchun Lan, Jingwen Niu, Lei Wang, Zhuoqing Qiao, Yu Zhang, Jing Ren, Bin Zhang, Liren Qian, Yehui Tan, Liping Dou, Yuhang Li, Liangding Hu

**Affiliations:** ^1^ Senior Department of Hematology, The Fifth Medical Centre of Chinese People’s Liberation Army General Hospital, Beijing, China; ^2^ Department of Hematology, The First Hospital of Jilin University, Changchun, China; ^3^ Department of Hematology, Chinese People's Liberation Army General Hospital, Beijing, China

**Keywords:** allogeneic haematopoietic stem cell transplantation, TP53 mutation, decitabine, conditioning regimen, myelodysplastic syndrome

## Abstract

Myelodysplastic syndrome (MDS) with TP53 mutations has a poor prognosis after transplantation, and novel therapeutic means are urgently needed. Decitabine (Dec) monotherapy has demonstrated improved overall response rates in MDS and acute myeloid leukaemia, although these responses were not durable. This study aimed to preliminary evaluate the efficacy of a Dec-containing allogeneic haematopoietic stem cell transplantation (allo-HSCT) preconditioning regimen in TP53-mutant MDS. Nine patients with TP53-mutant myelodysplastic syndromes received the decitabine-containing preconditioning regimen and subsequent myeloablative allo-HCT between April 2013 and September 2021 in different centres. At a median follow-up of 42 months (range, 5 to 61 months), the overall survival (OS) was 89% (8/9), progression-free survival (PFS) was 89% (8/9), and relapse incidence was 11.1%. The incidence of severe acute (grade III-IV) graft-versus-host disease (GVHD) was 22.2% (2/9) and that of chronic moderate-to-severe GVHD was 11.1% (1/9). The 1-year GVHD-free/relapse-free survival (GRFS) was 56% (5/9). In conclusion, we found real-world clinical data that supports the use of a Dec-containing preconditioning regimen before allo-HSCT for possible improved outcomes in TP53-mutant MDS patients; there is therefore an urgent call for an in-depth exploration of the involved mechanism to confirm these preliminary findings.

## Introduction

TP53, a tumour suppressor gene, plays a vital role in tumour inhibition through regulation of cellular senescence, apoptotic pathways, metabolism functions and DNA repair (1). TP53-mutant myelodysplastic syndrome (MDS) accounts for 5–10% of *de novo* MDS and 25–30% of therapy-related MDS, which is associated with worse outcomes (2–4). Allogeneic haematopoietic stem cell transplantation (allo-HSCT) is the only potentially curative treatment for MDS (5). However, TP53 mutations have also been shown to predict inferior outcomes for patients undergoing allo-HSCT owing to a high risk of relapse; the 3-year survival rate for these patients is still less than 20% (6, 7). Also, the median overall survival for these patients is typically less than 6 to 8 months (8). Even an escalated intensity of the conditioning regimen would fail to improve outcomes of patients with TP53-mutated MDS (5). Although BCL2 inhibitors have improved the treatment outcomes of patients with myeloid tumours (9, 10), AML patients with TP53 mutation still remain at high risk of drug resistance to venetoclax-based regimens (11, 12). Further, there are no US Food and Drug Administration–approved targeted therapies for the subset of patients with TP53 mutation. At the end of 2020, the leading targeted pharma-eprenatapopt (APR-246) missed the primary end point in phase 3 data, leaving us with no precision approaches for TP53-aberrant myeloid neoplasms (13). Nowadays, clinical trials regarding the nutlin analogs (MDM2 inhibitors), magrolimab (anti-CD47 antibody), and sabatolimab (TIM-3 antibody) are in the pharmacologic pipeline for treating TP53-mutant myeloid neoplasms (13). However, these novel targeted therapies may not eradicate the malignant hematological tumor stem cells, that is to say, only consolidative transplantation with curative intent possibly offers the highest chance of long-term survival for TP53-mutated patients. Therefore, the need for novel therapies pro and post transplantion is highlighted to improve the outcome of patients with TP53-mutated MDS. Given that TP53 clones are the major drivers of relapse for MDS patients, treatment before and after allo-HSCT to help patients get lower reduce recurrence and better OS may include: TP53 mutational clearance before transplantation (14), improvement of conditioning regimen during transplantation, and MRD (measurable residual disease)-adapted and maintenance strategies in the post-transplantation setting (15).

Decitabine (5-aza-2’-deoxycytidine), a cytosine analog, has promising clinical efficacy in the treatment of MDS, with evidence of hypomethylation and a favorable toxicity profile. Early studies have shown that 10-day courses of decitabine (Dec) resulted in an increased response rate in MDS with TP53 mutations, but rapid relapse resulted in no long-term benefit for patients (16). This study clarified that the short remission may result from incomplete clearance of leukaemia cells bearing the pathogenetically relevant driver mutations (16). Therefore, Dec shows unique therapeutic potential for TP53-mutation MDS but still needs to be improved. The addition of Dec to the conditioning regimen of allo-HSCT appears to be a reasonable improvement strategy, as allo-HSCT can achieve deeper remission with intensive chemotherapy and graft-versus-leukaemia (GVL) effect. The addition of Dec to the preconditioning regimen of allo-HSCT in our center exhibited good efficacy and safety for refractory or recurrent acute myeloid leukaemia (AML) (17). Additionally, a recent study also found that a Dec-containing preconditioning regimen may improve poor prognostic factors caused by poor-risk gene mutations in MDS and myeloproliferative neoplasms (18). Our study aimed to preliminary evaluate the efficacy of a Dec-containing allo-HSCT preconditioning regimen in TP53-mutant MDS. Therefore, 9 MDS patients with TP53 mutations who had undergone allo-HSCT were retrospectively analysed and compared.

## Materials and methods

### Study design and patients

Nine patients were selected in a consecutive manner between April 2013 and September 2021 in different centres (the Fifth Medical Centre of Chinese PLA General Hospital and the First Medical Centre of Chinese PLA General Hospital). During this period, study subjects who met the inclusion criteria (patients with TP53-mutant MDS who underwent allo-HSCT with decitabine-containing preconditioning regimen) were all included. All detected gene mutations were of somatic origin assessed by next generation sequencing (NGS). Detailed data were collected and recorded in standardised electronic forms and integrated by an experienced data administrator.

All patients were diagnosed according to the 2016 World Health Organization MDS Classification (19) and were evaluated using the IPSS-R risk categories (20). Allo-HSCT was performed immediately after the diagnosis of MDS in low- and high-risk categories, or upon indication for transplantation. Clinical data were acquired and collected prospectively, and retrospectively analysed. Approval for the study and sharing data with the coordinating institution was granted by the Fifth Medical Centre of the PLA General Hospital (No. KY-2019-12-57).

### Conditioning regimens

Myeloablative conditioning regimens were administered to all patients. Dec-containing regimens included Dec plus BU plus CY or Dec plus Flu plus Bu. Dec/BU/CY was administered as follows: Dec 25 mg/m^2^/d on day −11 to −9, BU 0.8 mg/kg q6h intravenously on day −7 to −4, and CY 60 mg/kg/day on day −3 to −2. Dec/Flu/Bu was administered as follows: Dec 25 mg/m^2^/d on day −11 to −9, FLU 30 mg/m^2^/d on day −8 to −4, and BU 0.8 mg/kg q6h intravenously on day −7 to −4.

### Prevention and management of GVHD

Patients who received HLA-matched transplants from siblings were administered cyclosporin A (CSA, 1.5 mg/kg/day intravenously until haematopoietic reconstitution, and thereafter orally to maintain blood concentrations between 150 and 200 ng/ml) and methotrexate (MTX, 15 mg/m^2^ on day +1, 10 mg/m^2^ on day +3, and day +6). Antithymocyte globulin (2.5 mg/kg/day on day -4 to -2), CSA, MMF (1.5 g/m^2^/day on days 0–28), and a brief course of MTX were administered to patients receiving HLA-mismatched transplants. CSA, a brief course of MTX, MMF, and balliximab (an anti-CD25 antibody) were administered to patients receiving unrelated grafts.

### Supportive care

All patients were treated in sterile laminar flow wards. To prevent viral infections and a *Pneumocystis carinii* infection, ganciclovir and trimethoprim sulfamethoxazole were routinely administered. Patients with and without invasive fungal infections were administered primary effective antifungal drugs and prophylactic fluconazole, respectively. Patients with platelet counts < 20 × 10^9^/L received transfusions of leukodepleted and irradiated platelets, while those with haemoglobin levels < 70 g/L received transfusions of leukodepleted and irradiated red blood cells. A combination of alprostadil, ursofalk, and heparin was used to prevent liver veno-occlusive disease.

### Definitions and assessments

The time from transplantation to any cause death or the last follow-up was defined as OS. Progression-free survival (PFS) was defined as the time from transplantation to the date of relapse or any-cause death. Relapse events were evaluated according to the International Working Group criteria (21). NRM and relative risk were defined based on standard criteria (22). The criteria defined by Przepiorka et al. were used to diagnose and grade acute GVHD (23). The National Institutes of Health scoring system was used to define chronic GVHD (21, 24). GVHD-free/relapse-free survival (GRFS) events were defined as grade 3–4 aGVHD or cGVHD requiring systemic immunosuppressive treatment, disease relapse, or any-cause death during the first 12 months after allogeneic HCT (25). The first three consecutive days with an absolute neutrophil count > 0.5 × 10^9^/L indicated neutrophil recovery. Platelet recovery was defined as the first seven days with an untransfused platelet count of > 20 × 10^9^/L.

### Statistical analysis

The Kaplan–Meier method was used to calculate survival curves, and the log-rank test was used to compare differences. Normally distributed data are presented as the mean ± standard deviation (SD), whereas non-normally distributed data are presented as the median. Statistical Package for the Social Sciences (SPSS; version 26.0) for Windows (SPSS, Chicago, IL) and GraphPad Prism version 9.2.0 were used for all statistical analyses (GraphPad Software, La Jolla, CA). A two-tailed p value of < 0.05 indicated statistical significance in all analyses.

## Results

### Characteristics of patients, diseases, and transplantation

The median patient age at the time of allo-HSCT was 48 years (range, 32-57 years) and the median follow-up time after transplantation was 42 months (range, 32 to 57 months). **Table 1** summarises the patient characteristics. None of these 9 patients received venetoclax as part of initial therapy. All patients underwent allo-HSCT after the 3-day Dec-containing myeloablative conditioning regimens and received peripheral blood-derived donor stem cells at a mean dose of 5.86 × 10^6^/L CD34+ cells. A total of six patients (67%) were diagnosed as MDS-RAEB (refractory anaemia with excess blasts) while the other three patients were diagnosed as MDS-MLD (multiple lineage dysplasia), MDS-RS (ring sideroblast)-MLD and MDS-5q- (MDS associated with isolated del(5q)) respectively. According to the IPSS-R for evaluating prognosis in MDS, four patients (44%) were in poor classification, and five patients (52%) were in very poor. **Supplementary Table 1** show the distribution of TP53 mutations and VAF in the training set. And the full set of mutations on each patient as determined by an NGS panel was provided in **Supplementary Table 2**.

**Table 1:** Patient and transplantation characteristics.

Characteristic	Value
**Age, (range, 32-57 years)**	48 (32∼57)
**Sex, n**	
**Male** **Female**	7 (78%) 2 (22%)
**MDS type, n**	
**MDS- MLD** **MDS-RS- MLD** **MDS-RAEB-1** **MDS-RAEB-2** **MDS-5q-**	1 (11**%**) 1 (11**%**) 4 (44**%**) 2 (22**%**) 1 (11**%**)
**Cytogenetics IPSS-R**	
**Good** **Int** **Poor** **Very-poor**	1 (11%) 2(22%) 2 (22%) 4 (44%)
**IPSS-R classification**	
**Poor** **Very poor**	4 (44%) 5 (56%)
**HCT-CI**	
**0** **1**	7 (78%) 2 (22%)
**Number of gene mutations**	
**≥3** **1–2**	6 (67%) 3 (33%)
**Treatment before allo-HSCT**	
**Chemotherapy** **Supportive care alone** **Immunosuppressive therapy** **Untreated**	2 (22%) 2 (22%) 1 (11%) 4 (44%)
**Bone marrow blasts before transplantation**	
**≤5%** **>5%**	3 (33%) 6 (67%)
**Donor/HLA type**	
**Matched related** **Mismatched related** **Matched unrelated** **Mismatched unrelated**	6 (67%) 1 (11%) 2 (22%) -
**Donor/recipient sex**	
**Female to male** **Others**	6 (66%) 3 (33%)
**Sex relationship**	
**Matched** **Unmatched**	5 (56%) 4 (44%)
**ABO compatibility**	
**Matched** **Major mismatched** **Minor mismatched** **Major and minor mismatched**	4 (44%) 3 (33%) 2 (22%) 0
**ABO blood type, n**	
**Matched** **Mismatched**	4 (44%) 5 (56%)
**Conditioning regimen, n**	
**Dec/BU/CY** **Dec/Flu/ Bu**	2 (22%) 7 (78%)
**ATG (g/kg)**	
**Yes** **No**	1 (11%) 8 (89%)
**Infused cells, mean dose (range)**	
**Mononuclear cells (×10^8^/kg)** **CD34+ (×10^6^/kg)**	10.55 (5.46-16.1) 5.85 (1.68-9.44)

MDS, myelodysplastic syndrome; RS, ring sideroblast; MLD, multiple lineage dysplasia; RAEB-1, refractory anaemia with excess blasts-1; MDS-5q-, MDS associated with isolated del(5q); IPSS-R, Revised International Prognostic Scoring System; HCT -CI, haematopoietic cell transplant-comorbidity index; Dec, decitabine; Bu, busulfan; Flu, fludarabine; Cy, cyclophosphamide; ATG, anti-thymocyte globulin.

### Engraftment and GVHD

**Table 2** lists the main events after allo-HSCT. All patients (9/9) had successful neutrophil engraftment after a median of 13 days (range, 12–18 days). At a median of 15 days (range, 12–20 days), platelet engraftment was completed in 100% of patients (9/9). In total, five patients (56%) developed aGVHD; of them, three patients (33%) and two patients (22%) had grade I–II and III–IV aGVHD, respectively. The most common site of aGVHD is skin (44%, 4/9), followed by digestive tract (11%, 1/9) and liver (11%, 1/9). The incidence of mild chronic GVHD was 67% (6/9) and that of moderate-to-severe cGVHD was 22% (2/9).

**Table 2:** Outcomes after allo-HSCT.

Outcome	Value
**Repopulation, n (%)**	9/9 (100%)
**Neutrophil repopulation (d), median (range)**	13 (12-18)
**Platelet repopulation (d), median (range)**	15 (12-20)
**aGVHD, n**	
**Grade III–IV** **Grade I–II** **Days post-transplantation, median (range)**	2 (22%) 3 (33%) 40 (1-100)
**cGVHD, n**	
**Mild** **Moderate-to-severe** **Days post-transplantation, median (range)**	4 (44%) 2 (22%) 240 (135-571)
**Outcomes of GVHD, n**	
**Remission** **No remission/dead**	8 0
**Relapse/progressive disease, n**	1 (11%)
**Death, n** **Relapse/progressive disease, n**	1 (11%) 1 (11%)

### Outcomes

**Figure 1:**
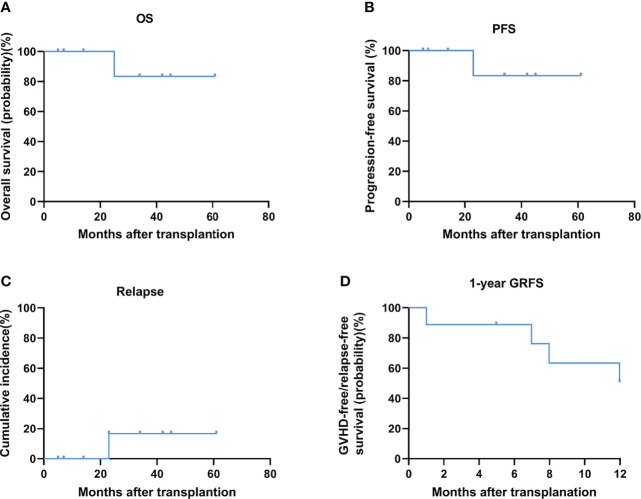
OS **(A)** and PFS **(B)** and relapse **(C)** and 1-year GRFS **(D)** after conditioning with decitabine at a median follow-up of 42 months after allo-HCT.

At a median follow-up of 42 months (range, 5 to 61 months), the overall survival (OS) was 88.9% (8/9) (**Figure 1A**) and the average survival time is as long as 31 months (the median survival time has not been determined). None of the 9 patients received any form of maintenance therapy after transplantation. The progression-free survival (PFS) was 88.9% (8/9) (**Figure 1B**), and relapse incidence was 11.1% (**Figure 1C**). Two months after the recurrence, the patient died of progressive disease. Of the 9 patients, there has been no non-relapse mortality death so far (NRM, 0). The incidence of severe acute (grade III-IV) graft-versus-host disease (GVHD) was 22.2% (2/9) and that of chronic moderate-to-severe GVHD was 11.1% (1/9). The 1-year GRFS was 55.6% (5/9) (**Figure 1D**). The specific clinical and cytogenetic characteristics of nine patients are described in **Tables 3** and **4**, and **Supplementary Tables 1**. All patients were diagnosed with MDS with a high or extremely high IPSS-R risk status. Although six patients of the nine patients received matched sibling donor allo-HSCT, the other three patients received alternative donor allo-HSCT. As we can see in **Table 3**, patient 5 and patient 7 achieved complete remission after receiving pre-transplant cytoreductive therapy. Because the significance of TP53 mutation as NGS-MRD marker for MRD detection in AML and MDS is not clear (26), the retesting of TP53 status before transplantation is not routinely carried out in our two centers.

**Table 3:** Characteristics of the nine patients.

	Patient 1	Patient 2	Patient 3	Patient 4	Patient 5	Patient 6	Patient 7	Patient 8	Patient 9
**Age**	48	37	32	54	38	48	37	57	53
**Sex**	M	M	M	M	M	F	F	M	M
**Diagnosis**	RAEB-1	MDS-RS-MLD	MDS-5q-	RAEB-1	RAEB-1	MDS-MLD	RAEB-1	RAEB-2	RAEB-2
**IPSS-R** **Classification**	Poor	Very poor	Poor	Very poor	Poor	Poor	Very poor	Very poor	Very poor
**TP53 VAF**	13.08%	49.55%	57.27%	76.90%	63.67%	5.63%	48.42%	48.78%; 45.12%	20.2%; 20.9%
**TP53 allelic state**	Undetermined	Multi-hit	Undetermined	Undetermined	Multi-hit	Multi-hit	Undetermined	Multi-hit	Multi-hit
**Treatment before** **transplant**	Untreated	IST	Supportive care	Untreated	Dec×1 (40 mg, d 1-5)	Supportive care	AZA×1 (100 mg, d 1-7)	Untreated	Untreated
**Disease status at transplant**	/	NR	/	/	CR1 (MRD+)	/	CR1 (MRD+)	/	/
**Donor type**	MSD	MSD	MUD	MSD	MSD	MSD	MUD	Haplo	MSD
**HLA matching**	10/10	10/10	10/10	10/10	10/10	10/10	10/10	5/10	10/10
**Donor sex**	F	F	F	M	M	F	F	M	F
**Grade III–IV** **aGvHD**	+	–	–	–	–	–	–	+	–
**moderate-or-severe** **cGvHD status**	+	–	–	–	+	–	–	–	–
**Relapse**	–	–	–	–	–	–	–	+	–
**NRM**	–	–	–	–	–	–	–	–	–
**OS (months)**	40	37	37	29	56	9	5	25	7

Dec, decitabine; MDS, myelodysplastic syndrome; RCUD, refractory cytopenia with uni-lineage dysplasia; RS, ring sideroblast; MLD, multiple lineage dysplasia; RAEB-1, refractory anaemia with excess blasts-1; MDS-5q-, MDS associated with isolated del(5q); IPSS-R, Revised International Prognostic Scoring System; IST, immunosuppressive therapy; AZA, Azacitidine; CR1, first complete remission; NR, non-remission; HLA, human leukocyte antigens; MSD, HLA-matched sibling donor; Haplo, HLA-haploidentical; MUD, HLA-matched unrelated donor; VAF, variant allele frequency; aGvHD, acute Graft versus Host Disease; cGvHD, chronic Graft versus Host Disease; NRM, non-relapse mortality; OS, overall survival.

**Table 4:** Cytogenetic characteristics of the nine patients.

Cytogenetic	
**Patient 1**	Abnormal karyotype: 46, XY, t(3,21)(q26.2;q22)[16]/ 46,XY, add(14)(q32), del(20)(q11.2)[1]/ 46,XY[3]
**Patient 2**	Complex karyotype; Monosomal karyotype: 45, XY, del(5q) (q15 q35), -6, der (18) (18pter->q21.1::?::6q11->6qter)[4]/ 44,idem,-3,add(8)(p11.2)[14]/ 43, idem,-3, add(8)(p11.2)-17[2]/ 46, XY, del(5q),(q15q35),+11,-18[2]/ 49, XY,del(5)(q15q35),+8,+11,+22[1] 5q del, trisomy 8 by FISH
**Patient 3**	Abnormal karyotype: 46, XY, del(5)(q22 q35)[18]/ 46,XY[3] 5q del by FISH
**Patient 4**	Complex karyotype; Monosomal karyotype: 44, X, -Y, -5, inv (8) (p11.2 q24.1), add (12) (p13) [16]/ 44, idem, del (7) (q21) [2]/ 45, idem, +8, inv (8) (p11.2q24.1) [3] 7q del, 20q del by FISH
**Patient 5**	Complex karyotype; Monosomal karyotype: 44, XY, -2, del(5)(q31q33), add(6)(q23), der(7) del(7)(q22q32) inv(7)(p13q22),+inv(11)(q13q25),-16, -17,20, +der(?)t(2;?)[10]/44,idem,+6,-add(6)(q23),-inv(11)(q13q25),+add(16)(p13.3)[4]/44, idem+6, add(6)(q23), -inv(11)(q13q25),+ 16,+ add(16)(p13.3), -19[1]/ 46, XY[8] 7q del, trisomy 8 by FISH
**Patient 6**	Complex karyotype: 47, XX, +8[4]/ 47, sl, del(5)(q31q33)[2]/ 48,sdl,+mar[2]/ 59<3n>,XXX,-2,-3,-5,+8,-9,-10,-11,-12,-14,-16, -17, add (6) (q23), -inv(11) (q13q25),+16,+ add(16)(p13.3), -19[1]/ 46, XY[8] 5q del, trisomy 8 by FISH
**Patient 7**	Complex karyotype: 46, XX, add (21) (q22) [11]/45-46, X, -X, add (21) (q22), +mar[cp2]/46, XX[2]
**Patient 8**	Complex karyotype: 41-45, XY, ?del(3)(p23),?Inv(3)(?P25?Q23),-5,?Dic(7;20)(p13;q11)[cp16]/42,XY,?Del(3)(q21),-5, -7,?Dic(7;20)(p13;q11),+8,+9,-16,?Del(17)(p11),-18,-19[1]/45,XY,?Inv(3)(?P25?Q23),-5,?Del(7) (p21),?Dic(7;20)(p13;q11),+22,+r[1]/41,XY,-1?Del(3)(p23),-5,?Del(7)(p13),-10,-18,-20[1]/46,XY[8] 5q del, 7q del,trisomy 8 by FISH
**Patient 9**	Abnormal karyotype: 49, XY, +8, t (11;19) (q23; p13), +18, +20[19] trisomy 8 by FISH

FISH, fluorescence in situ hybridisation.

## Discussion

TP53 is a key tumour suppressor gene involved in fundamental biological processes required for genomic stability and is recurrently mutated in a subgroup of MDSs and AML (8). It has been proven to be an adverse marker in the prognosis of AML and MDS patients and is associated with an increased proportion of blasts in the bone marrow, thrombocytopenia, complex karyotypes, and resistance to lenalidomide, hypomethylating agents, and allo-HSCT, all of which result in poor outcomes for MDS cases with TP53 mutations (2). Interestingly, recent clinical trials have demonstrated that patients with MDS harbouring TP53 mutations displayed favourable responses to treatment with Dec (16, 27). This case series assessed the efficacy of Dec-containing preconditioning regimen for MDS with TP53 mutation. Surprisingly, after transplantation, 8/9 of the MDS patients in the Dec group with TP53 mutations survived (median follow-up, 42 months; median overall survival, undefined). The PFS was 89% (8/9) and relapse incidence was 11.1%. Consistent with previous reports (17, 20, 28), it is seen that this scheme has good safety and feasibility. The incidence of severe acute (grade III-IV) graft-versus-host disease (GVHD) was 22.2% (2/9) and that of chronic moderate-to-severe GVHD was 11.1% (1/9). The 1-year GRFS, which really reflects the quality of life of patients after transplantation has also reached 56% (5/9). Our preliminary results showed such strategy is feasible and highly effective.

The recent findings suggest that the allelic state of TP53 is important for MDS prognosis, and this evaluation can be done by a combination of karyotyping and NGS. There is difference in the outcome when TP53-mutated patients had monoallelic mutations or had multiple hits (multi-hit) consistent with biallelic targeting (include three TP53-mutant subgroups: multiple mutations without deletion or copy-neutral loss of heterozygosity [cnLOH] affecting the TP53 locus, mutation(s) and concomitant deletion,mutation(s) and concomitant cnLOH). The evaluation of allelic state TP53 should be done by a combination of karyotyping (Conventional G-banding analysis, CBA) and NGS. TP53 multi-hit state predicted risk of death and leukemic transformation independently of the IPSS-R. Even worse, it is more difficult for multi-hit patients to get an effective response after treatments (29). In this multicentre study, 5/9 (Patients 2, 5, 6, 8 and 9) of the patients in our study had multi-hit TP53 mutations, which predict the risk of death and leukemic transformation independently of the IPSS-R (29). Unfortunately, due to the limitation of CBA testing technology, some of the patients in our data could not be tested for regions of copy-neutral loss of heterozygosity. Therefore, it is impossible to determine whether the other four cases (Patients 1, 3, 4 and 7) had multiple hits. Besides, Patient 3 and 4 have high VAF (>50%) which was strongly correlated with biallelic targeting and poor outcomes (30). Patient 3 was associated with del(5q), while both Patients 4 and 7 had complex karyotypes, and all of them are complicated with a high TP53 variant allele frequency. All of these factors have been identified as poor prognostic factors of TP53 mutation in different reports (7, 29, 31–35). Therefore, from the perspective of TP53 mutation frequency, accompanying chromosome karyotype, and allele status characteristics, at least 8/9 of patients(Except for patient 1) with TP53 mutations were comprehensively evaluated as patients with extremely poor prognostic outcomes.

The mechanism by which Dec treats TP53-mutant MDS is currently unclear. It has been reasonably suggested that Dec may increase the sensitivity of AML/MDS patients with TP53 mutations to treatment (16). However, canonical methylation signatures driven by TP53 mutations have not been identified to explain this sensitivity to Dec (16). Wang et al. found that patients who relapsed after allo-HSCT had significantly greater levels of methylation at TP53 than those in the non-relapse group (36). This indicated that TP53 may influence disease relapse through genetic mutations as well as epigenetic methylation pathways. Importantly, this result emphasises the prognostic importance of the methylation level of the TP53 gene and provides preliminary evidence to guide future research. Further studies on the correlation between methylation levels and prognosis of MDS patients with TP53 mutations will be helpful in understanding the unique mechanism of Dec in patients with TP53 mutations. A recent pre-clinical study *in vitro* suggested that busulfan (BU) plus cyclophosphamide (CY) plus Dec is synergistically cytotoxic to acute myeloid leukaemia (AML) cells regardless of the P53 status (P53-null or P53-wild-type) (31). They explained this sensitizing effect may owing to the molecular interactions between DNA alkylation and epigenetic modification events. Therefore, we can speculate that transplantation with a Dec-containing preconditioning regimen may further increase the depth of remission by chemotherapy synergy (37) and enhance the GVL effect (18, 38–40) on the basis of Dec sensitization, which needs to be confirmed by further studies.

To the best of our knowledge, there has no published data of azacytidine, another hypomethylating agent, as part of the preconditioning regimen before HSCT. Considering the extremely poor prognosis of patients with TP53 mutations, this paradigm of combination therapy also provides a novel strategy to reinforce the efficacy for other new drugs, such as MDM2 inhibitors, magrolimab, and sabatolimab. However, its rationality as part of a preconditioning regimen should be validated in preclinical studies first.

Although our report shows that there might be a subset of patients with TP53-mutant MDS, who derive benefit from Dec-containing conditioning regimen transplantation, it has limitations owing to its limited sample size. For example, the high rate of severe (grade III-IV) acute GVHD in our cohort (2/9, 22%) may also be caused by its statistical limitation of small sample size. However, given the low incidence of TP53 mutations in MDS, the high median age at onset (only a few patients are eligible for allo-HSCT), and the significantly increased detection rate of TP53 mutations in elderly patients (41), greater consideration for our findings is warranted. Similarly, the reason why the median age in our study was 48 years (lower than the median age of onset) is that only the patients who received allo-HSCT were included in our study, so relatively elderly patients were excluded. Besides, compared with the age of onset in western patients (more than 70 years old), the median age of onset in China is relatively younger (49-62 years old) (42–44). Lastly, the retrospective nature of our study might cause bias in interpretation of the data. So, further large-scale, prospective researches are required to corroborate the benefit of this novel therapy such that clinicians can apply this therapy for in an evidence-based manner.

In conclusion, our real-world clinical data support that Dec-containing preconditioning regimen before allo-HSCT is highly effective in TP53-mutant MDS patients and urgently call for mechanism and randomised prospective clinical studies to confirm these preliminary findings.

## Data availability statement

The datasets presented in this study can be found in online repositories. The names of the repository/repositories and accession number(s) can be found in the article/**Supplementary Material**.

## Ethics statement

The studies involving human participants were reviewed and approved by the ethics committee of the Fifth Medical Centre of the PLA General Hospital. The patients/participants provided their written informed consent to participate in this study.

## Author contributions

YW and YS performed the research and drafted the manuscript. JX was involved in the selection and management of the patient and in the manuscript review. LH and YL were involved in the identification, selection, and management of the patient and manuscript review. YT and LD provided Clinical samples of other centres. LQ and BZ put forward the modification suggestion. JH, NL, JC, BL, SL, JN, LW, ZQ, YZ, and JR collected and analysed the data. All authors contributed to the article and approved the submitted version.

## Funding

This work was supported by grants from the Program of Army Logistics Research (AWS17J010) and the National Natural Science Foundation of China (82100239).

## Conflict of interest

The authors declare that the research was conducted in the absence of any commercial or financial relationships that could be construed as a potential conflict of interest.

## Publisher’s note

All claims expressed in this article are solely those of the authors and do not necessarily represent those of their affiliated organizations, or those of the publisher, the editors and the reviewers. Any product that may be evaluated in this article, or claim that may be made by its manufacturer, is not guaranteed or endorsed by the publisher.

## References

[1] Molica M Mazzone C Niscola P de Fabritiis P. TP53 mutations in acute myeloid leukemia: Still a daunting challenge. Front Oncol (2020) 10:610820. doi: 10.3389/fonc.2020.610820 33628731 PMC7897660

[2] Jiang Y Gao SJ Soubise B Douet-Guilbert N Liu ZL Troadec MB. TP53 in myelodysplastic syndromes. Cancers (Basel) (2021) 13(21):5392. doi: 10.3390/cancers13215392 34771553 PMC8582368

[3] Della Porta MG Gallì A Bacigalupo A Zibellini S Bernardi M Rizzo E. Clinical effects of driver somatic mutations on the outcomes of patients with myelodysplastic syndromes treated with allogeneic hematopoietic stem-cell transplantation. J Clin Oncol (2016) 34:3627–37. doi: 10.1200/JCO.2016.67.3616 PMC6366344 27601546

[4] Garcia-Manero G Chien KS Montalban-Bravo G. Myelodysplastic syndromes: 2021 update on diagnosis, risk stratification and management. Am J Hematol (2020) 95:1399–420. doi: 10.1002/ajh.25950 32744763

[5] NCCN. Clinical practice guidelines in oncology-myelodysplastic syndromes(Version 3.2021)[DB/OL]. Available at: http://www.nccn.orgguidelinescategory_1.

[6] Lindsley RC Saber W Mar BG Redd R Wang T Haagenson MD. Prognostic mutations in myelodysplastic syndrome after stem-cell transplantation. N Engl J Med (2017) 376:536–47. doi: 10.1056/NEJMoa1611604 PMC5438571 28177873

[7] Hunter AM Sallman DA. Targeting TP53 mutations in myelodysplastic syndromes. Hematol Oncol Clin North Am (2020) 34:421–40. doi: 10.1016/j.hoc.2019.11.004 32089220

[8] Wang C Sallman DA. What are the prospects for treating TP53 mutated myelodysplastic syndromes and acute myeloid leukemia. Cancer J (2022) 28:51–61. doi: 10.1097/PPO.0000000000000569 35072374

[9] Sallman DA Komrokji R Vaupel C Cluzeau T Geyer SM McGraw KL. Impact of TP53 mutation variant allele frequency on phenotype and outcomes in myelodysplastic syndromes. Leukemia (2016) 30:666–73. doi: 10.1038/leu.2015.304 PMC7864381 26514544

[10] Deng J Wu X Ling Y Liu X Zheng X Ye W. The prognostic impact of variant allele frequency (VAF) in TP53 mutant patients with MDS: A systematic review and meta-analysis. Eur J Haematol (2020) 105:524–39. doi: 10.1111/ejh.13483 32621334

[11] Yoshizato T Nannya Y Atsuta Y Shiozawa Y Iijima-Yamashita Y Yoshida K. Genetic abnormalities in myelodysplasia and secondary acute myeloid leukemia: Impact on outcome of stem cell transplantation. Blood (2017) 129:2347–58. doi: 10.1182/blood-2016-12-754796 PMC5409449 28223278

[12] Aldoss I Pham A Li SM Gendzekhadze K Afkhami M Telatar M. Favorable impact of allogeneic stem cell transplantation in patients with therapy-related myelodysplasia regardless of TP53 mutational status. Haematologica (2017) 102:2030–8. doi: 10.3324/haematol.2017.172544 PMC5709102 28971906

[13] Patel SA Cerny J. TP53-mutant myelodysplastic syndrome and acute myeloid leukemia: The black hole of hematology. Blood Adv (2022) 6:1917–8. doi: 10.1182/bloodadvances.2021006580 PMC8941466 35090167

[14] Hunter AM Komrokji RS Yun S Al Ali N Chan O Song J. Baseline and serial molecular profiling predicts outcomes with hypomethylating agents in myelodysplastic syndromes. Blood Adv (2021) 5:1017–28. doi: 10.1182/bloodadvances.2020003508 PMC7903224 33591325

[15] Patel SA Lloyd MR Cerny J Shi Q Simin K Ediriwickrema A. Clinico-genomic profiling and clonal dynamic modeling of TP53-aberrant myelodysplastic syndrome and acute myeloid leukemia. Leuk Lymphoma (2021) 62:3348–60. doi: 10.1080/10428194.2021.1957869 34496723

[16] Welch JS Petti AA Miller CA Fronick CC O'Laughlin M Fulton RS. TP53 and decitabine in acute myeloid leukemia and myelodysplastic syndromes. N Engl J Med (2016) 375:2023–36. doi: 10.1056/NEJMoa1605949 PMC5217532 27959731

[17] Li Y Cheng L Xu C Chen J Hu J Liu N. A retrospective observation of treatment outcomes using decitabine-combined standard conditioning regimens before transplantation in patients with relapsed or refractory acute myeloid leukemia. Front Oncol (2021) 11:702239. doi: 10.3389/fonc.2021.702239 34504785 PMC8421765

[18] Cao YG He Y Zhang SD Liu ZX Zhai WH Ma QL. Conditioning regimen of 5-day decitabine administration for allogeneic stem cell transplantation in patients with myelodysplastic syndrome and myeloproliferative neoplasms. Biol Blood Marrow Transpl (2020) 26:285–91. doi: 10.1016/j.bbmt.2019.09.001 31494229

[19] Arber DA Orazi A Hasserjian R Thiele J Borowitz MJ Le Beau MM. The 2016 revision to the world health organization classification of myeloid neoplasms and acute leukemia. Blood (2016) 127:2391–405. doi: 10.1182/blood-2016-03-643544 27069254

[20] Wang QY Li Y Liang ZY Yin Y Liu W Wang Q. Decitabine-containing conditioning regimen for allogeneic hematopoietic stem cell transplantation in patients with intermediate- and high-risk myelodysplastic syndrome/acute myeloid leukemia: Potential decrease in the incidence of acute graft versus host disease. Cancer Manag Res (2019) 11:10195–203. doi: 10.2147/CMAR.S229768 PMC6900353 31824191

[21] Cheson BD Greenberg PL Bennett JM Lowenberg B Wijermans PW Nimer SD. Clinical application and proposal for modification of the international working group (IWG) response criteria in myelodysplasia. Blood (2006) 108:419–25. doi: 10.1182/blood-2005-10-4149 16609072

[22] Vardiman JW Thiele J Arber DA Brunning RD Borowitz MJ Porwit A. The 2008 revision of the world health organization (WHO) classification of myeloid neoplasms and acute leukemia: rationale and important changes. Blood (2009) 114:937–51. doi: 10.1182/blood-2009-03-209262 19357394

[23] Greenberg P Tuechler H Schanz J Sanz G Garcia-Manero G Solé F. Revised international prognostic scoring system for myelodysplastic syndromes. Blood (2012) 120(12):2454–65. doi: 10.1182/blood-2012-03-420489 PMC4425443 22740453

[24] Cruijsen M Hobo W van der Velden WJFM Bremmers MEJ Woestenenk R Bär B. Addition of 10-day decitabine to fludarabine/total body irradiation conditioning is feasible and induces tumor-associated antigen-specific T cell responses. Biol Blood Marrow Transplant (2016) 22(6):1000–8. doi: 10.1016/j.bbmt.2016.02.003 26860635

[25] Holtan SG Defor TE Lazaryan A Bejanyan N Weisdorf DJ. Composite end point of graft-versus-host disease-free, relapse-free survival after allogeneic hematopoietic cell transplantation. Blood (2015) 125:1333. doi: 10.1182/blood-2014-10-609032 25593335 PMC4335084

[26] Heuser M Freeman SD Ossenkoppele GJ Buccisano F Hourigan CS Ngai LL. 2021 Update on MRD in acute myeloid leukemia: a consensus document from the European LeukemiaNet MRD working party. Blood (2021) 138:2753–67. doi: 10.1182/blood.2021013626 PMC8718623 34724563

[27] Chang CK Zhao YS Xu F Guo J Zhang Z He Q. TP53 mutations predict decitabine-induced complete responses in patients with myelodysplastic syndromes. Br J Haematol (2017) 176:600–8. doi: 10.1111/bjh.14455 27984642

[28] Zhang C Chen XH Liu J Gao L Liu Y Gao L. Decitabine as a conditioning regimen in haploidentical stem cell transplantation for refractory acute myeloid leukaemia. J Clin Pharm Ther (2015) 40:336–8. doi: 10.1111/jcpt.12251 25825260

[29] Bernard E Nannya Y Hasserjian RP Devlin SM Tuechler H Medina-Martinez JS. Implications of TP53 allelic state for genome stability, clinical presentation and outcomes in myelodysplastic syndromes. Nat Med (2020) 26:1549–56. doi: 10.1038/s41591-020-1008-z PMC8381722 32747829

[30] Jivan Y Lane S. Not all TP53 mutations are equal. Hematologist (2021) 18(1).

[31] McGraw KL Cluzeau T Sallman DA Basiorka AA Irvine BA Zhang L. TP53 and MDM2 single nucleotide polymorphisms influence survival in non-del(5q) myelodysplastic syndromes. Oncotarget (2015) 6:34437–45. doi: 10.18632/oncotarget.5255 PMC4741464 26416416

[32] Mossner M Jann JC Nowak D Platzbecker U Giagounidis A Götze K. Prevalence, clonal dynamics and clinical impact of TP53 mutations in patients with myelodysplastic syndrome with isolated deletion (5q) treated with lenalidomide: results from a prospective multicenter study of the german MDS study group (GMDS). Leukemia (2016) 30:1956–9. doi: 10.1038/leu.2016.111 27133825

[33] Cumbo C Tota G Anelli L Zagaria A Specchia G Albano F. TP53 in myelodysplastic syndromes: Recent biological and clinical findings. Int J Mol Sci (2020) 21(10):3432. doi: 10.3390/ijms21103432 PMC7279310 32414002

[34] Flotho C Sommer S Lübbert M. DNA-Hypomethylating agents as epigenetic therapy before and after allogeneic hematopoietic stem cell transplantation in myelodysplastic syndromes and juvenile myelomonocytic leukemia. Semin Cancer Biol (2018) 51:68–79. doi: 10.1016/j.semcancer.2017.10.011 29129488

[35] de Witte T Bowen D Robin M Malcovati L Niederwieser D Yakoub-Agha I. Allogeneic hematopoietic stem cell transplantation for MDS and CMML: recommendations from an international expert panel. Blood (2017) 129:1753–62. doi: 10.1182/blood-2016-06-724500 PMC5524528 28096091

[36] Wang W Auer P Zhang T Spellman S Carlson KS Nazha A. Impact of epigenomic hypermethylation at TP53 on allogeneic hematopoietic cell transplantation outcomes for myelodysplastic syndromes. Transplant Cell Ther (2021) 27:659.e1–6. doi: 10.1016/j.jtct.2021.04.027 PMC8421055 33992829

[37] Valdez BC Tang X Li Y Murray D Liu Y Popat U. Epigenetic modification enhances the cytotoxicity of busulfan and4-hydroperoxycyclophosphamide in AML cells. Exp Hematol (2018) 67:49–59.e1. doi: 10.1016/j.exphem.2018.08.002 30102945 PMC6262883

[38] Cooley S Weisdorf DJ Guethlein LA Klein JP Wang T Marsh SG. Donor killer cell ig-like receptor b haplotypes, recipient HLA-C1, and HLA-c mismatch enhance the clinical benefit of unrelated transplantation for acute myelogenous leukemia. J Immunol (2014) 192:4592–600. doi: 10.4049/jimmunol.1302517 PMC4031316 24748496

[39] Mancusi A Ruggeri L Urbani E Pierini A Massei MS Carotti A. Haploidentical hematopoietic transplantation from KIR ligand-mismatched donors with activating KIRs reduces nonrelapse mortality. Blood (2015) 125:3173–82. doi: 10.1182/blood-2014-09-599993 25769621

[40] Zhang Z He Q Tao Y Guo J Xu F Wu LY. Decitabine treatment sensitizes tumor cells to T-cell-mediated cytotoxicity in patients with myelodysplastic syndromes. Am J Transl Res (2017) 9:454–65. PMC5340681 28337274

[41] Zhao WS Fan ZP Huang F Xu N Jiang QL Liu QF. [Clinical characteristics and prognosis of elderly patients with medium and high risk myelodysplastic syndrome]. Zhongguo Shi Yan Xue Ye Xue Za Zhi (2021) 29:840–6. doi: 10.19746/j.cnki.issn.1009-2137.2021.03.029. 34105481

[42] Wang W Wang H Wang XQ Lin GW. First report of incidence of adult myelodysplastic syndrome in China. Ann Hematol (2012) 91:1321–2. doi: 10.1007/s00277-011-1389-7 22194142

[43] Chen B Zhao WL Jin J Xue YQ Cheng X Chen XT. Clinical and cytogenetic features of 508 Chinese patients with myelodysplastic syndrome and comparison with those in Western countries. Leukemia (2005) 19:767–75. doi: 10.1038/sj.leu.2403688 15759035

[44] Wang XQ Ryder J Gross SA Lin G Irons RD. Prospective analysis of clinical and cytogenetic features of 435 cases of MDS diagnosed using the WHO (2001) classification: A prognostic scoring system for predicting survival in RCMD. Int J Hematol (2009) 90:361–9. doi: 10.1007/s12185-009-0403-5 19728027

