# Supplementary material for: Allogeneic haematopoietic stem cell transplantation with decitabine-containing preconditioning regimen in TP53-mutant myelodysplastic syndromes: A case study
Source: Front Oncol. 2022 Jul 18;12:928324. doi: 10.3389/fonc.2022.928324 (PMC9339648; doi:10.3389/fonc.2022.928324)
Supplement: Supplementary file 1 [file Table_1.docx]

| Gene Name | Transcript | DNA Mutation | Protein Mutation | Effect | Mean VAF | Del 17p/-17 | Corresponding patient |
| --- | --- | --- | --- | --- | --- | --- | --- |
| TP53 | NM_000546 | c.742C>T | p.R248W | non synonymous codon | 13.08 | No | Patient 1 |
| TP53 | NM_000546 | c.584T>A | p.I195N | non synonymous codon | 49.55 | Yes | Patient 2 |
| TP53 | NM_000546 | c.G818>A | p.R273H | non synonymous codon | 57.27 | No | Patient 3 |
| TP53 | NM_000546 | c.C799>T | p.R267W | non synonymous codon | 76.90 | No | Patient 4 |
| TP53 | NM_000546 | c.403T>C | p.C135R | non synonymous codon | 63.67 | Yes | Patient 5 |
| TP53 | NM_000546 | c.844C>G | p.Arg282Gly | non synonymous codon | 5.63 | Yes | Patient 6 |
| TP53 | NM_000546 | c.441delT | p.D148fs*22 | frameshift variant | 48.42 | No | Patient 7 |
| TP53 | NM_000546 | c.916C>T | p.R306* | stop gained | 48.78 | Yes | Patient 8 |
| TP53 | NM_000546 | c.994-1G>C | p.? | splice site | 45.12 | Yes | Patient 8 |
| TP53 | NM_000546 | c.742C>T | p.R248W | non synonymous codon | 20.2 | No | Patient 9 |
| TP53 | NM_000546 | c.536A>G | p.H179R | non synonymous codon | 20.9 | No | Patient 9 |

Supplementary Table 1. Distribution of TP53 mutations and VAF in the training set.

Supplementary Table 2. The full set of mutations on each patient as determined by an NGS panel.

| Case | mutations |
| --- | --- |
| Patient 1 | TP53, GATA2, TET2 |
| Patient 2 | TP53, BCOR, RELN, RBBP6 |
| Patient 3 | TP53, RUNX1, CSF3R |
| Patient 4 | TP53 |
| Patient 5 | TP53, C135R, ASXL1, SRSF2, TET2 |
| Patient 6 | ASXL1, TP53 |
| Patient 7 | BCOR, TET2, TP53 |
| Patient 8 | TP53 |
| Patient 9 | TP53, DNMT3A |
